# Novel Tripeptides as Tyrosinase Inhibitors: In Silico and In Vitro Approaches

**DOI:** 10.3390/ijms252413509

**Published:** 2024-12-17

**Authors:** Michał Dymek, Dawid Warszycki, Sabina Podlewska, Elżbieta Sikora

**Affiliations:** 1 Faculty of Chemical Engineering and Technology, Cracow University of Technology, Warszawska 24, 31-155 Kraków, Poland; michal.dymek@doktorant.pk.edu.pl; 2 Department of Medicinal Chemistry, Maj Institute of Pharmacology, Polish Academy of Sciences, Smętna 12, 31-343 Kraków, Poland

**Keywords:** tyrosinase inhibitors, tripeptides, molecular modeling, melanogenesis

## Abstract

Tyrosinase is a key enzyme responsible for the formation of melanin (a natural skin pigment with ultraviolet-protection properties). However, some people experience melanin overproduction, so new, safe, and biocompatible enzyme inhibitors are sought. New tripeptide tyrosinase inhibitors were developed using molecular modeling. A combinatorial library of tripeptides was prepared and docked to the mushroom tyrosinase crystal structure and investigated with molecular dynamics. Based on the results of calculations and expert knowledge, the three potentially most active peptides (CSF, CSN, CVL) were selected. Their in vitro properties were examined, and they achieved half-maximal inhibitory concentration (IC_50_) values of 136.04, 177.74, and 261.79 µM, respectively. These compounds attach to the binding pocket of tyrosinase mainly through hydrogen bonds and salt bridges. Molecular dynamics simulations demonstrated the stability of the peptid–tyrosinase complexes and highlighted the persistence of key interactions throughout the simulation period. The ability of these peptides to complex copper ions was also confirmed. The CSF peptide showed the highest chelating activity with copper. The 3-(4,5-dimethylthiazol-2-yl)-2,5-diphenyltetrazolium bromide (MTT) assay confirmed that none of the test tripeptides showed cytotoxicity toward the reconstructed human epidermis. Our results indicated that the developed tripeptides were non-toxic and effective tyrosinase inhibitors. They could be applied as raw materials in skin-brightening or anti-aging cosmetic products.

## 1. Introduction

Skin is the outer barrier of the human body. It is responsible for the protection of the body from external factors, such as air pollution, toxic chemicals, pathogenic microorganisms, or ultraviolet (UV) radiation [1]. The frequency of exposure to the sun can trigger oxidative stress and inflammation or lead to cell damage or tumor formation [2]. One of the defense mechanisms of the body against UV radiation is the production of the dark pigment melanin [3,4]. Melanin is formed in specialized cells called melanocytes, and its most common varieties are eumelanin and pheomelanin. The protective role of melanin is significant, but in some cases, the pigment is overproduced. The phenomenon is manifested by the formation of melanin clusters on the skin and the development of freckles or age spots, also known as “hyperpigmentation changes” [5,6,7]. To deal with this problem, it is necessary to intervene in one of the stages of melanogenesis.

Tyrosinase is the key enzyme that manifests its activity, especially at the beginning of melanin synthesis [8]. It is responsible for catalyzing the oxidation of tyrosine or levodopa (L-DOPA) to dopaquinone, which is then converted to melanin. The use of substances that bind to the active site of this enzyme will stop or slow down the formation of pigment and, thus, reduce the intensity of skin changes [7,9].

In the food and cosmetic industries, tyrosinase inhibitors of natural [10,11,12,13] and synthetic origin [14,15,16] are used, including resveratrol [17], arbutin [18], kojic acid [19], azelaic acid [20], or L-ascorbic acid [21]. Mixtures of substances are also used, for example, in the form of plant extracts, with or without the isolation of individual compounds responsible for the inhibition [22]. However, the adverse effects associated with their use (proven toxicity, mutagenic or carcinogenic potential, as well as causing secondary skin inflammation) have led to restrictions in their applications [23,24,25,26]. Several countries have banned the use of hydroquinone for skin treatments. Some restrictions have been introduced in the use of kojic acid or arbutin [27]. Therefore, it is necessary to develop non-toxic and biocompatible molecules with a skin-brightening effect. Among all tyrosinase inhibitors, peptides deserve special attention. Thanks to the multitude of possible combinations of amino acids, peptides can stimulate cells to synthesize biomolecules, increase transport of other ingredients to the skin, or modulate the action of enzymes. Peptides with tyrosinase-inhibitory properties have been identified but not described thoroughly. Thaha et al. [28] indicated that peptides obtained from natural raw materials with the amino-acid sequence VWWW, IRW, and VKAGFAWTANQQLS could be antioxidants and substances preventing the darkening of products caused by tyrosinase-catalyzed oxidation. Feng et al. [29] studied a tripeptide with the sequence FPY, present in *Juglans regia* L. (walnut), which is a tyrosinase inhibitor. Peptides with the sequences NYRRE and RHAKF obtained from Chinese quince have also been tested [30] for tyrosinase-inhibiting activity. Active dipeptides with the sequences IR, LK, and VY have also been identified [31]. Hsiao et al. [32] developed new peptide inhibitors based on pharmacophore modeling, resulting in two cysteine-containing tripeptides: CRY and RCY. CRY was characterized as having the best tyrosinase-inhibitory properties among test compounds.

Attempts have also been made to modify peptide molecules with moieties that increase the stability of the peptide–protein complex based on non-peptide inhibitors. J.-M. Noh and colleagues [33] described the amides of kojic acid and tripeptides obtained by solid-phase synthesis. They showed a stronger inhibitory effect than pure kojic acid (reference substance), similar to the combination of thiosemicarbazone with tripeptides with the sequences FFY, FWY, and FYY [34].

Often, test peptides are isolated from natural resources, but synthetic compounds have also been used [35]. A thorough analysis encompassing all possible combinations of tripeptides and their potential inhibitory activity against tyrosinase has not been carried out. A method that permits the screening of large databases or combinatorial libraries is called “virtual screening”. It is used widely in the development of small molecule-active substances with a description of their interaction in the binding pocket of the enzyme [32]. In silico methods significantly reduce the time needed to find potentially effective substances, eliminating the need for tedious syntheses and, thus, reducing costs. Molecular modeling allows for the initial characterization of compounds, exploring vast chemical space, and finding the molecular mechanisms of action [19,36,37].

In this work, new tripeptide tyrosinase inhibitors were studied. A virtual combinatorial library of tripeptides was generated and docked to the crystal structure of tyrosinase. All docked compounds were classified based on the presence and quality of interactions with the binding pocket, the value of the scoring function, and expert knowledge. The three most promising tripeptides were synthesized on demand, and their ability to reduce tyrosinase activity was determined experimentally. Additionally, the best peptides were subjected to a 1000 ns molecular dynamics simulation, including the mapping and timeline representation diagrams showing protein–ligand contacts.

## 2. Results and Discussion

### 2.1. In Silico Study

#### 2.1.1. Sequence Alignment

**Figure 1:**
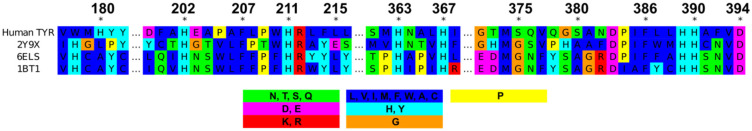
Multiple sequence alignment of tyrosinases from different species: humans (Human TYR); *Agaricus bisporus* (2Y9X); *Malus domestics* (6ELS); and *Ipomoea batatas* (1BT1). The figure shows a fragment of the sequence preserving the amino acids located near the active site and histidine residues responsible for copper coordination. The colors of cells correspond to the types of amino acids: green (polar); purple (acidic); red (basic); blue (hydrophobic); turquoise (polar and aromatic); orange and yellow (amino acids breaking the secondary structure). The asterisk (*) indicates the location of the specific amino acid number mentioned above.

Sequence alignment is a useful tool to identify protein fragments (including enzymes) that are similar at the structural level. The manipulation of human tyrosinase presents inherent challenges; its crystal structure is not available, so it is necessary to carry out homology modeling or use other enzymes that are most structurally similar. Entire sequences of four enzymes, from *A. bisporus* (2Y9X), *M. domestics* (6ELS), *I. batatas* (1BT1), and human tyrosinase, were aligned using Clustal Omega. Figure 1 illustrates the MSA of tyrosinases from different species only for selected segments relevant to the active site of tyrosinase to ensure better readability. Twenty-seven identical residues were identified (including six histidine residues), which were responsible for the coordination of copper ions in the active center (which is crucial for the catalytic ability of tyrosinases) [38]. *A. bisporus* (2Y9X), *M. domestics* (6ELS), and *I. batatas* (1BT1) showed 20.12%, 18.98%, and 19.72% identity with human tyrosinase, respectively.

Tyrosinase from *A. bisporus* (2Y9X) is used as a substitute for human tyrosinase in virtual screening [39,40,41]. Among the aligned sequences, mushroom tyrosinase showed the highest similarity to human tyrosinase, so it was selected for molecular modeling. An additional advantage is the possibility of testing compounds on the same enzyme in in vitro inhibition tests and in silico studies. Nonetheless, it is important to note that sequence identity does not always equate to structural similarity [42]. While the primary structure, or the amino acid sequence, may be similar, this does not necessarily imply resemblance in secondary, tertiary, or quaternary structures.

#### 2.1.2. Molecular Docking and Virtual Screening of Tripeptides

Virtual screening is a powerful and relatively inexpensive tool for selecting compounds with potential specific biological activity. Searching for tyrosinase inhibitors enables the rapid testing of thousands of potential tripeptide combinations, thereby leading to the identification and selection of the most promising candidates [43,44].

In the traditional approach (high-throughput screening), to make such a selection, it would be necessary to synthesize or purchase 8000 tripeptides and then test them in vitro. This process would be time-consuming but also expensive, outweighing the potential profit. However, thanks to virtual screening, these limitations can be avoided, thereby accelerating the research process significantly and reducing expenses [44].

After appropriate preparation of *A. bisporus* tyrosinase (PDB ID: 2Y9X) and the ligands, molecular docking was carried out. The test peptides were ranked according to the docking score (Table 1).

**Figure 2:**
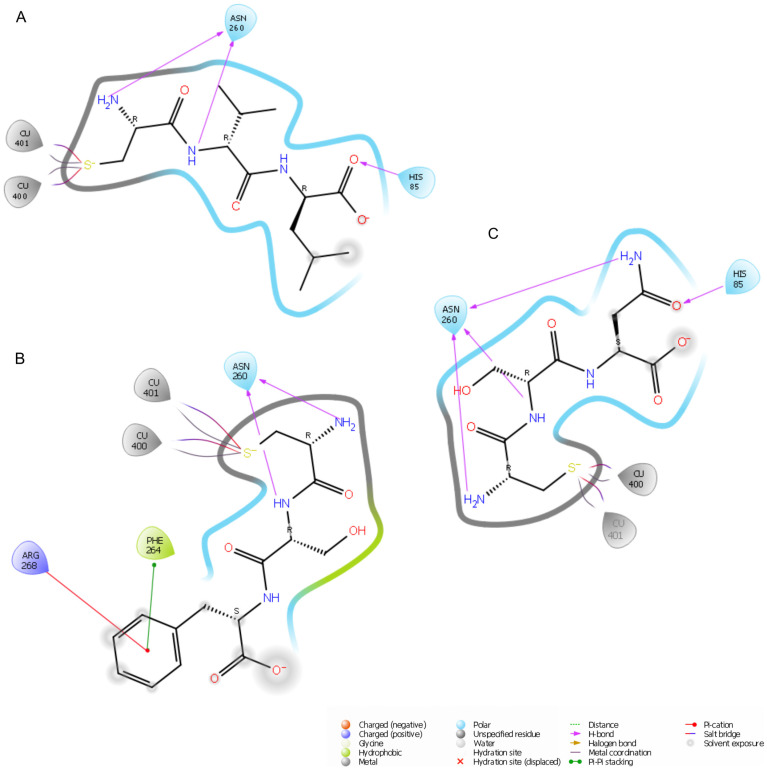
Binding interactions for CVL (**A**), CSF (**B**), and CSN (**C**) tripeptides within the 2Y9X binding site.

The docking score of the best-fit conformation of the CVL tripeptide was −7.989 (the lower the value, the better the fit). It was the lowest value among the test compounds, followed by the CSF tripeptide and CSN tripeptide, with docking stores of −7.542 and −7.519, respectively. Analysis of the docked poses (Figure S1 from Supplementary Materials) indicated that each tripeptide was cysteine-directed through a tight passage to the copper atoms located in the active center. The CSF tripeptide and CSN tripeptide, sharing two identical amino acids, had a consistent arrangement in the binding site, but the oxygen atom of the serine carbonyl group in CSN had a different orientation. For CSF, it was oriented to His85. For CSN, it was oriented in the Phe264 region. Phenylalanine in CSF occupied a cavity on the surface of tyrosinase, arranging the benzene ring between the methyl groups of Val248 and the aromatic ring of Phe264. The hydrophobic nature of the residue may have further contributed to the stabilization of the complex. For CVL, leucine occupied the site on the opposite side of Val248 from CSF and was exposed to the solvent. It was also stabilized by interaction via the oxygen atom with His85, which also coordinated one of the copper atoms (Figure S1 and Figure 2).

A thorough investigation of the molecular interactions between a tripeptide and the protein provides valuable insights into the complex stability. Figure 2 shows the binding interactions between the test peptides and the amino acids in the active pocket. All compounds formed salt bridges with copper atoms (Cu 400 and 401) via the thiol group, which was in an ionized form under physiological docking conditions (pH = 7.40). Copper is a key element of tyrosinase and is directly responsible for its catalytic activity [45]. Consequently, creating a strong bond could impede its accessibility to the substrate involved in melanogenesis. In addition, each peptide made contact with certain histidine residues: His61; His85; His94; His259; His263; and His296. Only CSF showed no interaction with His244. The CVL peptide (A) had a hydrogen bond (H-bond) of the carbonyl group of leucine with His85 (distance = 1.84 Å). Additional H-bonds were present between the amino group (2.22 Å) and carbonyl group (2.09 Å) of cysteine and Asn260. There was a hydrophobic interaction between Val248 and the hydrocarbon chain of leucine. The binding mode of the CSF tripeptide showed π–π stacking interactions between the benzene ring of phenylalanine in the peptide and Phe264, as well as additional stabilization by π–cation interactions with Arg268. The NH group of serine and NH_2_ of cysteine formed H-bonds with Asn260 with a length of 2.05 Å and 2.26 Å, respectively. Four H-bonds were observed for the docked CSN(C) peptide: three between Asn260 and the NH_2_ groups of cysteine, asparagine, and NH of serine (2.23 Å, 2.69 Å, and 2.37 Å, respectively), as well as between His85 and the carbonyl oxygen of the asparagine residue of CSN (2.51 Å). Our simulations indicated that, apart from the electrostatic attraction with histidine groups, the formation of an H-bond with Asn260 was typical for all tested N-terminal cysteine peptides. This finding is in agreement with the work of Tien-Sheng et al. [46], in which they investigated the effectiveness of tyrosinase inhibition by dipeptide combinations. N-terminal cysteine-containing peptides show stronger interactions than C-terminal analogs. Studies based on quantitative structure–activity relationships have confirmed the higher activity of peptides with N-terminal cysteine. Also, hydrophobic interactions with Val283 occur in peptides with two and three amino acids [47].

#### 2.1.3. Molecular Dynamic Simulations

MD is a powerful method applied to the analysis of interactions between active compounds and targets along with their temporal fluctuations. MD uses approximations based on Newtonian physics, allowing for the simulation of mutual movements of atoms in large biological molecules, which would be impossible or very time-consuming using quantum methods that require enormous computational resources [48,49]. Thanks to MD, it is possible to visualize conformational changes, ligand binding, or protein folding in a strictly controlled environment, also in the presence of water molecules or ions, which are often crucial for the activity of the enzyme [50].

**Figure 3:**
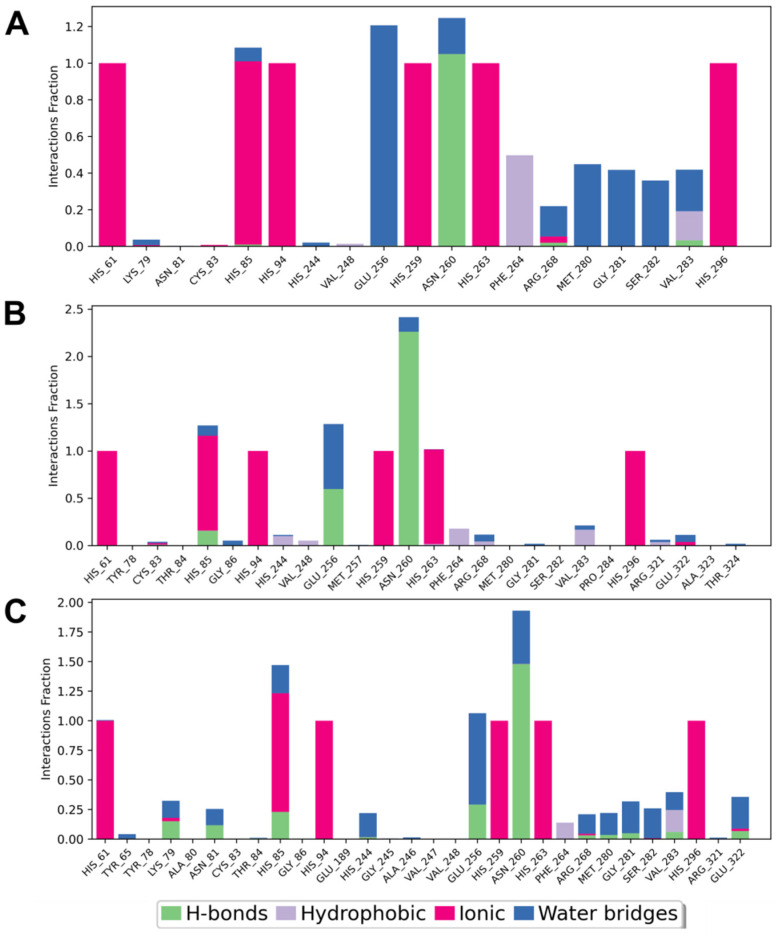
Mapping of tyrosinase–ligand contacts for the selected tripeptides CVL (**A**), CSF (**B**), CSN (**C**) as a result of 1000-ns MD simulation. The symbols of amino acids forming contacts during the analysis are placed on the horizontal axis, and the corresponding interaction fraction is placed on the vertical axis. The colors in the charts correspond to different types of interactions: H-bonds (green); hydrophobic (purple); ionic (pink); or water bridges (blue). The interaction fraction represents the proportion of time-specific interactions maintained throughout the trajectory snapshots.

Molecular-docking calculations were complemented by undertaking MD simulations using the Desmond package (Schrödinger Release 2024-4; D.E. Shaw Research, New York, NY, USA; Maestro-Desmond Interoperability Tools, Schrödinger). To better understand intermolecular interactions, diagrams showing protein–ligand contact were prepared for each of the docked peptides from 1000-ns MD simulations. These diagrams provided information on the type and frequency of interactions between the individual amino acids of the enzyme and the inhibitor and how they changed during the simulation. As key factors influencing the stability of the complex, H-bonds, hydrophobic and ionic interactions, as well as water bridges, were monitored during this experiment (Figure 3). Moreover, the last frame of each simulation was taken for further consideration as a starting point of the MMGBSA analysis, which allowed for defining predicted binding energy.

All test compounds showed similar binding modes with *A. bisporus* tyrosinase in vitro (Figure 3). All simulated tripeptides showed the presence of characteristic interactions with numerous histidine components previously indicated in molecular-docking studies: His61; His85; His94; His244; His259; His263; and His296. These were mainly ionic interactions (although His85 also showed contacts in the form of H-bonds and water bridges). Similarly, for each peptide, the presence of H-bonds and (to a greater extent for CSN and CVL) water bridges with Asn260, as well as water bridges with Glu256, were observed.

Considering individual compounds, for CVL, ionic interactions occurred with Arg268, hydrophobic interactions with Phe264 and Val283, limited H-bonds with Arg268 and Val283, and water bridges with Lys79, Arg268, Met280, Gly281, Ser282, and Val283. In the case of CSF peptide, there were ionic interactions with Cys83 and Glu322, hydrophobic interactions with Phe264, Val248, Arg268, Val283, and Arg321, H-bonds with Glu256, and water bridges with Gly86, Glu256, Arg268, Gly281, Val283, Glu322, and Thr324. For CSN, there were practically no ionic interactions with amino acids other than the previously mentioned histidine. H-bonds were present with Lys79, Asn81, Glu256, Arg268, Met280, Gly281, Val283, and Glu322. Hydrophobic interactions were formed with Phe264 and Val283, and water bridges were formed with Tys65, Lys79, Asn81, Arg268, Met280, Gly281, Ser282, Val283, and Glu322.

**Figure 4:**
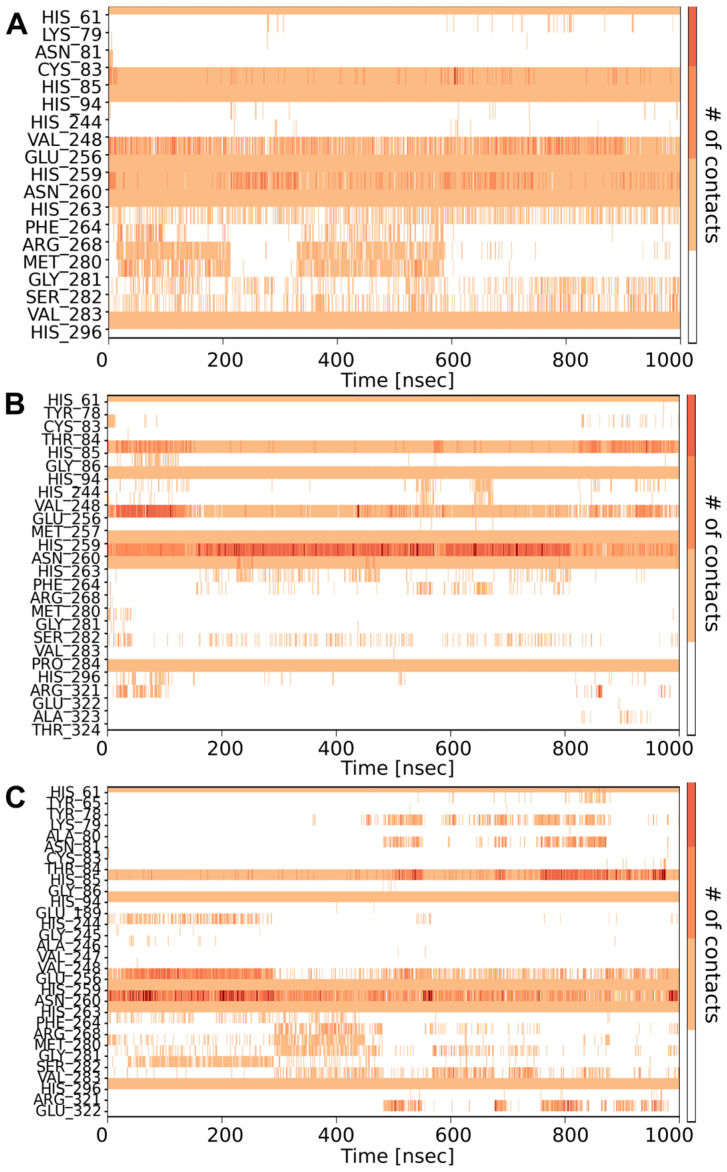
Timeline representation of protein–ligand contacts for CVL (**A**), CSF (**B**), and CSN (**C**) tripeptides. Total number of specific protein–ligand contacts is shown in the top panels of each compound as dark-blue lines. The vertical axis of the bottom panels lists the amino acids of tyrosinase interacting with peptide inhibitors, whereas the horizontal axis is the time scale of the simulation (1000 ns). Darker shades of orange correspond to more abundant active pocket–inhibitor contacts in each trajectory frame.

Timescale analysis (Figure 4) indicated that all tripeptides interacted with histidine groups present in the binding pocket throughout the simulation. Over the full range of simulations, the CVL tripeptide was bound to Glu256 and Asn260. Contacts with Phe264, Arg268, Met280, Gly281, Ser282, and Val283 also seemed to be significant (though they did not occur during the whole duration of the simulation). For the CSF peptide, unlike in static docking tests, the interaction with Phe264 occurred only for a certain time of the experiment, similar to contacts with Cys83, Gly86, Val248, Arg268, Gly281, and Glu322. Numerous and stable contacts with Glu256 and Asn260 seemed to be crucial for activity, occurring until the end of the simulation with the highest number of contacts among all tripeptides. In the case of CSN peptide, stable contacts appeared with Glu256 and Asn260; the compound interacted slightly less frequently with Arg268, Met280, Gly281, Ser282, Val283, and Glu322.

**Figure 5:**
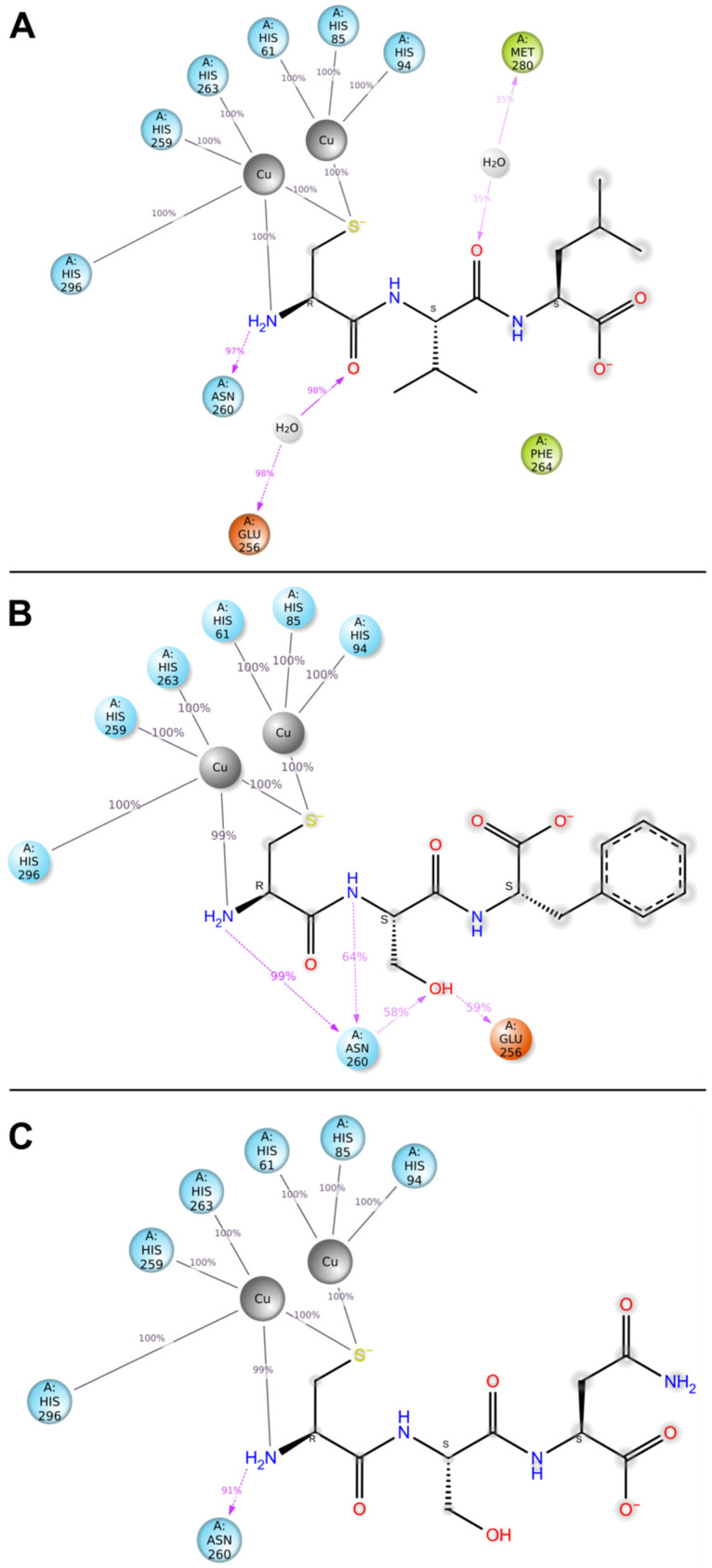
Interactions between ligand atoms and tyrosinase residues for CVL (**A**), CSF (**B**), and CSN (**C**) tripeptides (schematic). The contacts shown occurred >30% of the simulation time. The colors of enzyme residues correspond to their chemical nature: polar (blue); negatively charged (orange); and hydrophobic (green). Copper atoms are represented as gray spheres. Percent values indicate the frequency of the interaction during analyses.

Diagrams containing the structures of the analyzed compounds (Figure 5) also indicated that the interaction between the thiol group of cysteine (present in all three peptides) and the copper ions of tyrosinase persisted for 100% of the selected time trajectory interval. This is crucial in terms of the effectiveness of inhibitors because, as indicated above, copper is responsible for the catalytic activity of the enzyme. A comparison of the types of interactions occurring in molecular docking for ≥30% of the simulation time and MD results indicated some differences in both approaches (Table 2). A common feature was the presence of salt bridges with copper ions and an H-bond with Asn260. In the case of the CVL peptide, there was no H-bond with His85, yet water bridges with Glu256 and Met280 appeared. CSF tripeptide in MD did not show long-term π–π or π–cation interactions, but it formed an additional H-bond with Glu256, which was not shown by the other two test compounds. Moreover, unlike CVL and CSN, it formed more H-bonds with Asn260 than indicated by molecular docking. Finally, CSN in MD did not interact via H-bonds with His85. The observed differences in results for molecular docking and MD for tripeptides were likely attributable to adjustments to computational conditions to better simulate realistic environments (protein mobilization, presence of solvent) or the dynamic nature of the compound within the binding pocket. These factors resulted in the continuous formation and disruption of contacts.

Assessing the efficacy, safety, and potential active-substance interactions relies heavily on the careful determination of absorption, distribution, metabolism, and excretion (ADME) parameters. This determination constitutes a key phase in the development of novel active compounds and employs computational and empirical approaches. The use of in silico methods, as in the case of virtual screening, allows for faster and low-cost estimation of these parameters, thereby shortening the time needed to select appropriate compounds. One of the most frequently used rules for estimating druglikeness is Lipinski’s RO5. In general, it states that a bioactive substance cannot have more than one violation of the following criteria: number of H-bond donors (nHBD) ≤ 5; number of H-bond acceptors (nHBA) ≤ 10; molecular weight (MW) ≤ 500; or logP ≤ 5. Otherwise, the bioactive substance may exhibit poor absorption or permeation [51]. The results of the prediction of RO5 parameters are shown in Table 3. All compounds had an MW < 500 and were hydrophilic (logP < 0). The peptides chosen on the basis of molecular docking met Lipinski’s RO5, although CSN showed one rule violation (nHBD > 5).

Melanogenesis occurs in the melanocytes residing in the skin epidermis. Hence, it is necessary to deliver tyrosinase inhibitors through the epidermis. The extent of skin permeation can be estimated using the skin permeability coefficient (Kp) based on MW and logP [52]. Due to the hydrophilicity of our tripeptides, potential diffusion through the skin would probably be low. Hence, it seems reasonable to encapsulate the peptides in carriers (e.g., liposomes [53]) to enhance the permeation rate.

### 2.2. Tyrosinase-Inhibition Assay and IC_50_ Determination

To verify the results obtained during molecular docking and MD, in vitro tests were carried out on tyrosinase from the cultivated mushroom *A. bisporus*. During testing, the ability of peptides and kojic acid (reference substance) to inhibit the enzyme was studied. IC_50_ values were determined to compare the activity of the compounds. The test results are presented in Table 3.

As predicted using computational methods, all developed tripeptides showed tyrosinase inhibition but differed in activity. The CSF tripeptide demonstrated the lowest IC_50_ value (136.04 ± 4.02 µM), which was 1.9- and 1.3-fold lower than for CVL (261.79 ± 3.64 µM) and CSN (177.74 ± 2.66 µM) peptides, respectively. The lipophilicity of the compound and, therefore, its solubility in water (all substances were highly soluble under the experimental conditions), expressed as logP, did not seem important for anti-melanogenetic properties. Interaction analysis for docked peptides indicated that, for the CSF peptide, there were exclusively π–π stacking interactions with Phe264 and π–cation interactions with Arg268. However, taking into account long-term (1000 ns) MD simulations, multiple contacts of H-bonds and water bridges with Glu256 and Asn260 may also have been responsible for the higher CSF activity, as indicated above (Table 2).

The reference substance, kojic acid, had a lower IC_50_ value (45.14 ± 1.52 µM) than the tripeptides in our study, similar to the one obtained by Peng and coworkers: 48.05 ± 3.28µM [54]. Kojic acid is one of the most frequently used tyrosinase inhibitors in the cosmetic industry [9], but some restrictions have been introduced in its use [27]. The inhibition parameters may vary depending on the protocol used and the purity of the enzyme. For kojic acid, IC_50_ values have been reported in the range of 10–300 µM [55]. However, kojic acid presents a significantly different chemical structure, so it is rational to compare the results with other peptide compounds. The obtained IC_50_ values for the best-characterized tripeptide, CSF, were much lower than those of the FPY peptide (3.22 ± 0.09 mM) [29], IQSPHFF heptapeptide (4 mM) [56], SWY peptide (272 µM) [57], or ECGYF (0.46 mM) [58]. Only in the case of the CRY tripeptide was the IC_50_ lower in comparison with our results (6.16 µM). However, other studies were conducted using L-tyrosine instead of L-DOPA as a substrate, which affected the results [32]. Molecular docking indicated that this peptide coordinated copper ions in the active site of tyrosinase via the thiol group, similar to the peptides discussed in this work. Under similar conditions, using L-tyrosine, dipeptides containing cysteine at the N-terminus were characterized by IC_50_ values in the range of 2.0–55.8 µM. In their case, a similar interaction profile could also be observed, including π–cation interactions with His244, Asn260, Glu256, or Phe264 [46].

No direct correlation was observed between the docking scores (expressed as Glide score) and IC_50_. However, a trend was noted when using MM/GBSA binding energy. The CSF peptide exhibited the highest binding energy value (−18.75 kcal/mol), which corresponded to its lowest IC_50_ value, indicating the highest inhibitory efficiency. Similarly, the CSN and CVL peptides showed binding energy values of −15.75 and −11.11 kcal/mol, respectively, aligning with their higher IC_50_ values and lower tyrosinase inhibition efficiency. MM/GBSA utilizes data from molecular dynamics (MD) simulations, enabling the evaluation of protein–ligand interactions across various conformations. This method also accounts for the influence of water and environmental factors on these interactions, which are critical for binding in biological systems. In contrast, docking typically overlooks these effects or addresses them in a highly simplified manner [59].

**Figure 6:**
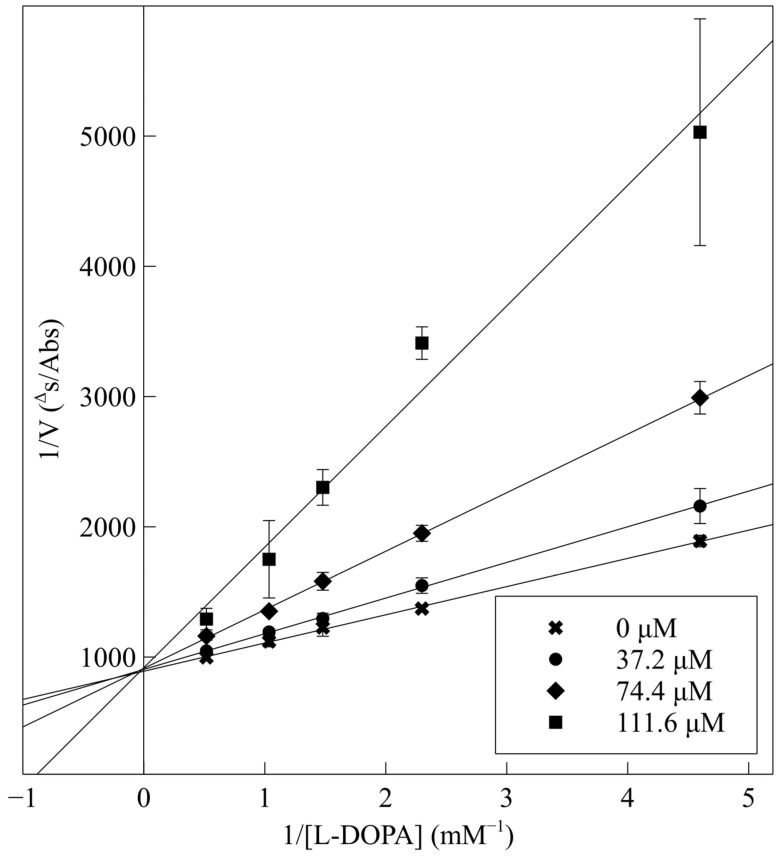
Lineweaver–Burk plot for the inhibition of mushroom tyrosinase activity by the CSF peptide at different concentrations of the inhibitor (0, 37.2, 74.4, and 111.6 µM).

The Lineweaver–Burk plot is a useful tool for analyzing enzyme kinetics and identifying the type of inhibition. It allows for a deeper understanding of the mechanisms of action of enzymes and their inhibitors. A kinetic analysis was carried out on the CSF peptide, which exhibited the lowest IC_50_ value among the test peptides. The plot depicting the relationship between 1/V and 1/[L-DOPA] is presented in Figure 6.

The four lines plotted for various peptide concentrations (ranging from 0 to 111.6 µM) exhibited different slopes but intersected at a single point on the vertical axis. This finding indicated that the V_max_ values remained consistent across all series, suggesting that CSF acted as a competitive inhibitor. It did not form an enzyme–substrate complex but, instead, was bound solely to the free enzyme. This observation provides additional experimental confirmation of the molecular modeling we conducted. The inhibitor occupied the same active site on the enzyme as L-DOPA, thereby preventing further substrate transformation.

### 2.3. CuChA

Due to the key importance of copper ions in tyrosinase activity, analysis of the interactions between copper and potential chelators may contribute to understanding the functioning of inhibitors. The results of the CuChA assay are shown in Table 4.

All test peptides showed similar CuChA but were significantly (*p* < 0.01) lower than that of the reference (EDTA), which is a strong and widely used metal-complexing agent. Under test conditions, the CSF peptide showed the highest activity (63.70%), followed by CSN (56.49%) and CVL (48.05%). Significant differences (*p* < 0.05) were observed between CVL and CSF samples. The in vitro tyrosinase-inhibition assay for *A. bisporus* also indicated that the CSF tripeptide had the lowest IC_50_ value. Probably, the crucial role is assumed by cysteine, located at the N-terminus of each tripeptide. Molecular docking confirmed that strong interactions in the form of salt bridges were present in the tyrosinase-binding pocket between the thiol group of cysteine and copper ions. This electrostatic attraction is believed to affect the chelating activity of peptides. Moreover, studies have shown that Cu(II) ions in the presence of cysteine could be reduced to Cu(I), which was then complexed by excess cysteine [60]. This phenomenon may further explain the ability of cysteine-containing peptides to block copper ions. Furthermore, all three test compounds were peptides with low MW, which is consistent with observations made in other studies. Torres-Fuentes et al. [61] suggested that peptides of small molecular size present the best CuChA, as well as compounds containing many histidine units, which is also confirmed by how copper is coordinated within tyrosinase. Peptides with MW of 0.6–1.3 kDa also show very good CuChA, up to 94.43% [62]. Small differences in the activity of the test peptides resulted from the types of the remaining two amino acids (apart from cysteine), which might contribute to copper chelation in different ways. Amino acids located in the middle of a chain or at its C-terminus can interact with copper with varying strength through nitrogen atoms (amide group) or oxygen atoms (hydroxyl or carboxyl groups), which are electron-pair donors [63,64,65,66]. Some studies have indicated that copper ions can be chelated by phenylalanine, which occurs at the N- or C-terminus of the peptide [67].

### 2.4. Cytotoxicity of Tripeptides

To assess potential cytotoxic properties, tests were undertaken on the RHE model. In the colorimetric test, the amount of formosan produced was determined spectrophotometrically. Cell survival after use of the test peptides is presented in Table 5.

Results related to the positive control (PC), which contained SDS and had a destructive effect on cells (cell viability = 6.2 ± 0.6%), and the negative control (NC), which contained PBS and was inert to cell viability (100.0 ± 0.8%). No cytotoxic potential was observed for any test peptides because cell survival was >50% in each case [68,69]. The CSN peptide achieved the highest cell viability (102.2 ± 2.4%), which also indicated the slight proliferative properties of the peptide. CSF and CVL peptides had lower values, 90.7 ± 13.7% and 77.8 ± 19.9%, respectively, and showed no significant difference with the NC result. Despite the peptides under investigation being novel and so far unattained compounds, the observed variations in results could be explained by considering the impact of amino-acid composition, which are crucial components in cell cultures [70,71,72]. Asparagine (present in the CSN tripeptide) can affect the growth and survival of cells, especially those deprived of glutamine, by maintaining protein production [73,74]. Asparagine also regulates the uptake of serine [75], which has antioxidant and cytoprotective effects [76]; this probably contributed to the overall result.

## 3. Materials and Methods

### 3.1. Materials

Mushroom tyrosinase (T3824-25KU), L-DOPA, kojic acid, and pyrocatechol violet were purchased from Millipore Sigma (Burlington, MA, USA). Peptides were synthesized on demand by Novazym (Poznań, Poland) using solid-phase synthesis. Potassium dihydrogen phosphate (POCh, Gliwice, Poland), sodium hydrogen phosphate (POCh), methanol (Chempur, Piekary Slaskie, Poland), and acetonitrile (Chempur) were of analytical grade. The in vitro EpiDerm™ Skin Irritation Test (EPI-200-SIT) kit was purchased from MatTek (Bratislava, Slovak Republic). The deionized water used in all assays was additionally filtered through the Milli-Q™ system (Merck Millipore, Waltham, MA, USA).

### 3.2. In Silico Study

#### 3.2.1. Multiple Sequence Alignment (MSA)

At the time of preparation of this manuscript, the crystal structure of human tyrosinase was not available. Tyrosinase sequences from the cultivated mushroom (*Agaricus bisporus*; Protein Database (PDB) ID: 2Y9X), apple (*Malus domestica*; PDB ID: 6ELS), sweet potato (*Ipomoea batatas*; PDB ID: 1BT1), and humans were chosen for our study. To investigate the relationship between proteins, the MSA of enzymes was performed, and their identity was compared. MSA was carried out using Clustal Omega via UniProt (www.uniprot.org/, accessed on 30 July 2024) [77].

#### 3.2.2. Preparation of Protein Structure

The three-dimensional structure of tyrosinase 2Y9X was prepared using the Protein Preparation Wizard [78] in Maestro (Schrödinger Release 2024-4: Schrödinger, New York, NY, USA). The protein was pre-processed by removing holmium atoms and tropolone molecules. Subsequently, bond orders were assigned; hydrogen atoms were added, and zero-order bonds to metals (copper atoms) were created using Prime [79,80]. Het states were generated using Epik [81,82] (Schrödinger Release 2024-4: Schrödinger, New York, NY, USA) for pH = 7.40. All water molecules were removed from the structure.

#### 3.2.3. Docking Studies and Virtual Screening of Tripeptides

Virtual screening of possible tripeptide combinations was undertaken to select the most effective tyrosinase inhibitors. A combinatorial library of 20 natural amino acids combined into 8000 tripeptides in the simplified molecular-input line-entry system (SMILES) was downloaded from data provided in the literature [83]. Initially, the set of ligands was subjected to the LigPrep tool in Maestro (Schrödinger Release 2024-4: Schrödinger, New York, NY, USA). The three-dimensional structure of test compounds was optimized, generating ionized states for pH = 7.40, using the OPLS4 force field [84]. Metal binding states were added, and the “include the original state” option was disabled.

The receptor grid was generated using Glide [85,86,87,88] (Schrödinger Release 2024-4: Schrödinger, New York, NY, USA) centered on the location of the two copper atoms in the binding pocket. The default parameters were a van der Waals radius scaling factor of 1.0, a partial charge cutoff of 0.25, and no additional constraints. Ligands were docked using default settings (standard precision, flexible ligand sampling, nitrogen inversions, ring conformations sampling, bias sampling of torsions for all predefined functional groups, and added Epik state penalties to the docking score). Post-docking minimization was undertaken for 1 pose per ligand. The best three tripeptides were selected according to the number and type of interactions within the binding site, scoring function, and expert knowledge.

In addition, the parameters for Lipinski’s rule of five (RO5) were determined using SwissADME (www.swissadme.ch/, accessed on: 30 July 2024) [89].

#### 3.2.4. Molecular Dynamics (MD)

MD simulations were carried out using the Desmond Molecular Dynamics System [90] (Schrödinger Release 2024-4; D.E. Shaw Research, New York, NY, USA; Maestro-Desmond Interoperability Tools, Schrödinger) for the three potential inhibitors selected in the previous step. Molecular dynamics simulations started from the ligand’s docking pose within the binding pocket. Copper atom preparation followed the same protocol as docking. Tyrosinase–peptide complexes obtained during molecular docking were minimized using the OPLS4 force field. Complexes were analyzed for their stability and conformational flexibility during the 1000-ns simulation at the physiological concentration of NaCl (150 μM) at 300 K, using the constant normal pressure and lateral surface area of membranes and constant temperature (NPAT) ensemble. A simulation time of 1000 ns was recorded with a 100 ps trajectory interval, with the following parameters: elapsed 0.0; energy 1.2; RESPA integrator time step [fs] bonded 2.0; Nose–Hoover chain thermostat method with 1.0 ps relaxation time, and Martyna–Tobias–Klein barostat method with relaxation time of 2.0 ps and constant area coupling style. The Coulombic cutoff radius of 9.0 Å was used. An orthorhombic simulation box of size 10 Å × 10 Å × 10 Å was prepared, and the TIP4P solvent model was applied.

#### 3.2.5. Molecular Mechanics General Born Surface Area Analysis (MM/GBSA)

MMGBSA calculations were performed in Prime software [79,80] under the default settings (solvation model VSGB, force field OPLS4, sampling method—minimizing and using ligand partial charges). Structures of protein and peptide were taken from the last frame from MD trajectory. Only no strain values were further considered.

### 3.3. Assay to Measure Mushroom Tyrosinase Inhibition and Kinetic Analysis

Tyrosinase-inhibition tests were carried out to confirm the predicted whitening properties and determine the half-maximal inhibitory concentration (IC_50_) of the new tripeptides. A procedure described by Channar et al. [91] with slight modifications was applied.

Solutions of phosphate-buffered saline (PBS; 67 mM) at pH 6.8, L-DOPA (5 mM) in PBS, and mushroom tyrosinase in PBS (52.5 units/mL) were prepared. The assay was carried out in a 96-well plate by mixing 50 μL of the peptide solution (PBS for the control sample), 80 μL of PBS, and 40 μL of tyrosinase solution. The final concentration of studied peptides was in the range of 0–1190 μM for each active substance. Kojic acid was used as a reference substance. The plate was incubated at 25 °C for 5 min, and then, 40 μL of L-DOPA solution or, in the case of a blank sample, 40 μL of PBS, was added. Absorbance at 475 nm was measured 30 min after initiation of the reaction using a plate reader (Infinite 200Pro; Tecan, Männedorf, Switzerland). Each measurement was made in triplicate. The IC_50_ value was the concentration of the active compound, which led to 50% inhibition of the enzyme. It was obtained from the alignment between the inhibitor concentration and dose–response curve [32] and was calculated using the AAT Bioquest calculator (www.aatbio.com/tools/ic50-calculator/, accessed on: 30 July 2024).

On the basis of the IC_50_ value, the most potent inhibitor was selected for kinetic analysis. The mode of inhibition was determined using the Lineweaver–Burk plot of 1/V against 1/[S], where V was the initial velocity of the reaction, and [S] was the L-DOPA concentration in the range of 0.22 mM to 1.93 mM, as an average of three independent experiments. Maximal initial velocity was determined from the initial linear portion of absorbance up to 5 min after L-DOPA addition. The peptide concentrations used in the assay were 0, 37.2, 74.4, and 111.6 μM.

### 3.4. Determination of Copper-Chelating Activity (CuChA)

The CuChA of test tripeptides was determined using the method described by Kubglomsong et al. [92]. Briefly, 10 μL of each peptide sample (5 mM), 280 μL of sodium acetate buffer (50 mM; pH 6.0), 10 μL of CuSO_4_·5H_2_O (1 mg/mL), and 6 μL of pyrocatechol violet (4 mM) were mixed in a 96-well plate for 5 min. Absorbance at 632 nm was measured 30 min after reaction initiation using a plate reader (Infinite 200Pro; Tecan, Männedorf, Switzerland). Ethylenediaminetetraacetic acid (EDTA) was used as the positive sample. The control sample contained 10 μL of water instead of the peptide. The CuChA was calculated according to Equation (1).

(1) $$I\%=\frac{A_{B}-A_{P}}{A_{B}}\cdot 100\%$$

where A_B_ is the absorbance of the control sample, and A_P_ is the absorbance of the peptide sample.

Statistical analysis was conducted using the PSPP software (FSF, Boston, MA, USA), version 1.6.2. One-way analysis of variance (ANOVA) was carried out. For comparisons that revealed significant differences between groups (*p* < 0.05), Tukey’s post hoc test was applied to determine pairwise differences.

### 3.5. Evaluation of the Cytotoxicity of Tripeptides

Our aim was to assess the safety of the developed tripeptides or their potential cytotoxicity. Hence, an in vitro skin-irritation test was undertaken on the EpiDerm-200-reconstructed human epidermis (RHE) model. The procedure described by Kandarova et al. [93] was used. Before experimentation, all tissues were visually inspected for defects or flaws. Their preparation consisted of a 60-min and an 18-h pre-incubation periods. Each time, incubation was undertaken in a sterile apparatus at 37 °C and 95% relative humidity in an atmosphere of 5% CO_2_. The next day, 30 μL of a peptide solution (111.6 μM) was applied to cells. PBS of pH 7.40 was used as a negative control, and 5% sodium dodecyl sulfate (SDS) was used as a positive control. The contact time of tissues with test compounds was 60 min. Then, they were washed thoroughly with PBS and transferred to a fresh medium, which was replaced after a 24-h incubation. After a total time of 42 h, cells were transferred to 3-(4,5-dimethylthiazol-2-yl)-2,5-diphenyltetrazolium bromide (MTT) solution and incubated for 3 h. After transferring samples to a fresh plate, the formed compound (formosan) was extracted for 2 h with isopropanol on a shaker (120 rpm). The analysis was carried out employing a UV-visible spectrophotometer (Nanocolor™; Macherey-Nagel, Düren, Germany) at 570 nm using isopropanol as a blank. Cell viability was calculated according to Equation (2):

(2) $$Vpep\%=\frac{A_{PEP}}{A_{N}}\cdot 100\%$$

where V_pep%_ is cell viability; A_PEP_ is the mean absorbance of peptide-exposed samples, and A_N_ is the mean absorbance of the negative control. Data were statistically analyzed using the method described in Section 3.4.

## 4. Conclusions

Using molecular modeling, three new tripeptides, with the sequences CVL, CSF, and CSN, were developed as potential tyrosinase inhibitors. The activity of these peptides was verified in vitro and showed promising enzyme inhibitory results, as well as copper chelating properties. Kinetic analysis of tyrosinase inhibition for the CSF peptide (with the lowest IC_50_ value) suggested that it was a competitive inhibitor, binding to the free enzyme and preventing the substrate from reacting. This finding was also confirmed in in silico studies: all compounds strongly occupied the active site of tyrosinase, creating several characteristic interactions, including salt bridges via cysteine. The stability of the formed complexes was confirmed by MD simulations for 1000 ns. Additionally, MM/GBSA analysis provided a better correlation with the experimental results than docking studies. Finally, an assay for the viability of RHE cells showed that the described compounds did not show cytotoxicity. Our results indicated that these new tripeptides were non-toxic and effective tyrosinase inhibitors. They could be applied as potential raw materials in skin-brightening or anti-aging cosmetic products.

## Figures and Tables

**Table 1:** Scores and binding energies of three most potent tripeptide tyrosinase inhibitors docked into the active site of the enzyme (C = cysteine; V = valine; L = leucine; S = serine; F = phenylalanine; N = asparagine).

Peptide Structure	Peptide Name	Glide Gscore [kcal/mol]	Docking Score [kcal/mol]	MMGBSA Binding Energy [kcal/mol]
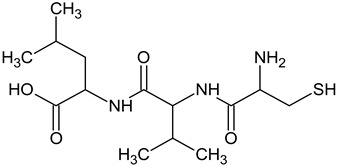	CVL Cys-Val-Leu	−8.415	−7.989	−11.11
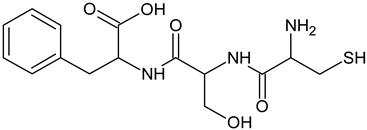	CSF Cys-Ser-Phe	−7.966	−7.542	−18.75
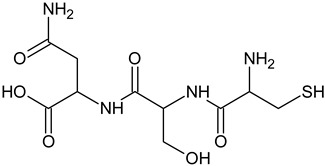	CSN Cys-Ser-Asn	−7.943	−7.519	−15.75

**Table 2:** Comparison of the peptide–tyrosinase residue interactions occurring in molecular docking (DOC) and molecular dynamics (MD) results. In the case of MD, the indicated interactions occurred for ≥30% of the simulation time.

Amino Acid	CVL		CSF		CSN	
	DOC	MD	DOC	MD	DOC	MD
Cu/Cu	Salt bridge	Salt bridge	Salt bridge	Salt bridge	Salt bridge	Salt bridge
His85	1× H-bond	-	-	-	1× H-bond	-
Glu256	-	Water bridge	-	H-bond	-	-
Asn260	2× H-bond	1× H-bond	2× H-bond	3× H-bond	3× H-bond	1× H-bond
Phe264	-	-	Π-Π	-	-	-
Arg268	-	-	Π-cation	-	-	-
Met280	-	Water bridge	-	-	-	-

**Table 3:** Experimental IC_50_ values and RO5 parameters for test compounds. * represents one violation of Lipinski’s RO5.

Peptide	IC_50_ ± SD [µM]	Glide Gscore [kcal/mol]	MMGBSA Binding Energy [kcal/mol]	MW [g/mol]	logP	nHBD	nHBA	logKp [cm/s]	RO5
CVL	261.79 ± 3.64	−8.415	−11.11	333.45	−0.13	4	5	10.44	Yes
CSF	136.04 ± 4.02	−7.966	−18.75	355.41	−0.92	5	6	10.61	Yes
CSN	177.74 ± 2.66	−7.943	−15.75	322.34	−3.37	6	7	12.63	Yes *
Kojic acid	45.14 ± 1.52	-	-	142.11	−0.16	2	4	7.62	Yes

**Table 4:** Copper-chelating activity for test tripeptides.

Peptide	CVL	CSF	CSN	EDTA
CuChA [%]	48.05 ± 7.98 ^a,b^	63.70 ± 5.82 ^a,c^	56.49 ± 5.87 ^d^	88.77 ± 1.93 ^b,c,d^

The same subscripts indicate significant differences between samples (^a^—*p* < 0.05; ^b,c,d^—*p* < 0.01).

**Table 5:** Viability of RHE cells for test tripeptides. Values are an average of three independent measurements.

Peptide	CVL	CSF	CSN	PC	NC
Viability [%] ± SD	77.8 ± 19.9 ^a,b,c^	90.7 ± 13.7 ^d^	102.2 ± 2.4 ^a,e^	6.2 ± 0.6 ^c,d,e,f^	100.0 ± 0.8 ^b,f^

The same subscripts indicate significant differences between samples (^a,b^—*p* < 0.05; ^c,d,e,f^—*p* < 0.0001).

## Data Availability

Data are contained within this article and Supplementary Materials.

## Supplementary Materials

The following supporting information can be downloaded at https://www.mdpi.com/article/10.3390/ijms252413509/s1.
