# Supplementary material for: Novel Tripeptides as Tyrosinase Inhibitors: In Silico and In Vitro Approaches
Source: Int J Mol Sci. 2024 Dec 17;25(24):13509. doi: 10.3390/ijms252413509 (PMC11677486; doi:10.3390/ijms252413509)
Supplement: Supplementary file 1 [file ijms-25-13509-s001.zip › ijms-3300850-supplementary.pdf]

Supplementary Materials

Novel Tripeptides as Tyrosinase Inhibitors: *In Silico* and *In Vitro* Approaches

Michał Dymek[1,*], Dawid Warszycki[2], Sabina Podlewska[2], Elżbieta Sikora[1,*]

[1] Faculty of Chemical Engineering and Technology, Cracow University of Technology, Warszawska 24, 31-155 Kraków, Poland

[2] Department of Medicinal Chemistry, Maj Institute of Pharmacology, Polish Academy of Sciences, Smętna 12, 31-343 Kraków, Poland

* Correspondence: elzbieta.sikora@pk.edu.pl

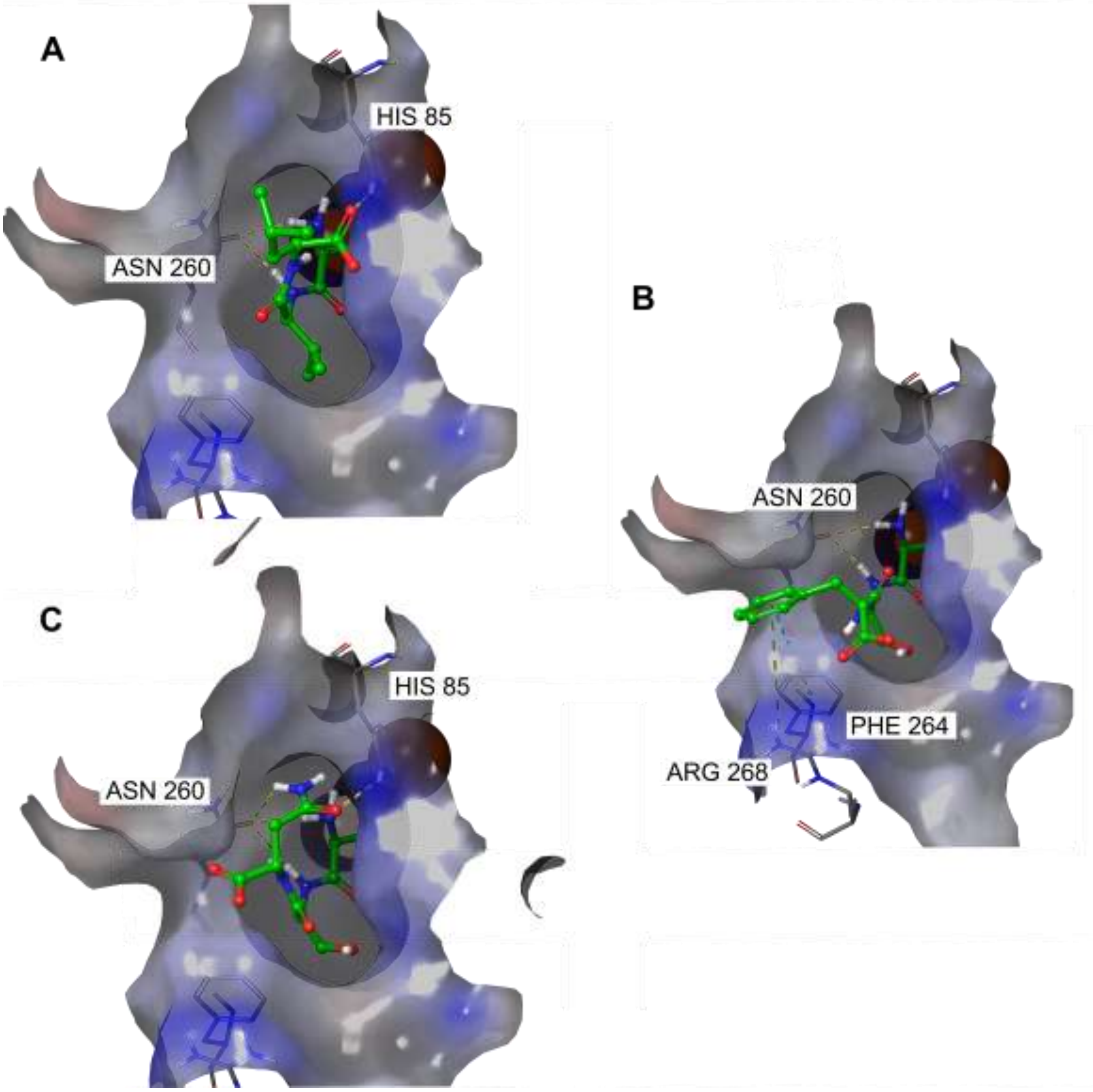

Figure S1. Docking poses of CVL (panel A), CSF (B), and CSN (C) tripeptides in the 2Y9X binding site. The blue of the protein surface represents more positive electrostatic potential. The atoms of the docked compounds are colored as follows: green (carbon), white (hydrogen), red (oxygen), blue (nitrogen), and yellow (sulfur). Brown spheres represent copper atoms in the active center. Only the amino acids in tyrosinase that form the key interactions have been labeled. Dashed lines indicate different types of interactions: hydrogen (yellow), π–π (blue), and π–cation (green).
